# Isolation and effect of *Trichoderma citrinoviride* Snef1910 for the biological control of root-knot nematode, *Meloidogyne incognita*

**DOI:** 10.1186/s12866-020-01984-4

**Published:** 2020-10-02

**Authors:** Haiyan Fan, Meiling Yao, Haiming Wang, Di Zhao, Xiaofeng Zhu, Yuanyuan Wang, Xiaoyu Liu, Yuxi Duan, Lijie Chen

**Affiliations:** 1 Nematology Institute of Northern China, College of Plant Protection, Shenyang Agricultural University, Shenyang, China; 2 Analytical and Testing Center, Shenyang Agricultural University, Shenyang, China; 3 College of Biological Science and Technology, Shenyang Agricultural University, Shenyang, China; 4 College of Sciences, Shenyang Agricultural University, Shenyang, China

**Keywords:** Fungal bioagent, Nematode disease management, Plant growth promotion, Tomato

## Abstract

**Background:**

Root-knot nematode is one of the most significant diseases of vegetable crops in the world. Biological control with microbial antagonists has been emerged as a promising and eco-friendly treatment to control pathogens. The aim of this study was to screen and identify novel biocontrol agents against root-knot nematode, *Meloidogyne incognita*.

**Results:**

A total of 890 fungal isolates were obtained from rhizosphere soil of different crops and screened by nematicidal activity assays. Snef1910 strain showed high virulence against second stage juveniles (J2s) of *M. incognita* and identified as *Trichoderma citrinoviride* by morphology analysis and biomolecular assay. Furthermore, *T. citrinoviride* Snef1910 significantly inhibited egg hatching with the hatching inhibition percentages of 90.27, 77.50, and 67.06% at 48, 72, and 96 h after the treatment, respectively. The results of pot experiment showed that the metabolites of *T. citrinoviride* Snef1910 significantly decreased the number of root galls, J2s, and nematode egg masses and J2s population density in soil and significantly promoted the growth of tomato plants. In the field experiment, the biocontrol application showed that the control efficacy of *T. citrinoviride* Snef1910 against root-knot nematode was more than 50%. Meanwhile, *T. citrinoviride* Snef1910 increased the tomato plant biomass.

**Conclusions:**

*T. citrinoviride* strain Snef1910 could be used as a potential biological control agent against root-knot nematode, *M. incognita*.

## Background

Root-knot nematode (RKN) is one of the most seriously damaging plant-parasitic nematode in the world threatening to the growth and production of more than 5500 plants, including vegetable crops and weeds [1–3]. Among them, *Meloidogyne incognita*, *M. arenaria*, *M. javanica*, and *M. hapla* are the four main root-knot nematodes that have been reported. *M. incognita* is the most serious with 51% of the affected crops and the highest distribution proportion among these four species [4]. With rapidly increased developing protected agriculture, high-value crops, such as tomato (*Solanum lycopersicum* cultivar L-402 susceptible to *M. incognita*), are severely damaged by *M. incognita* and has led to severe losses in China [5]. Currently, chemical and physical measures have limited use for RKN disease management. Furthermore, as the nematicides are associated with serious environmental problems. Therefore, the safe and effective strategies for management for *M. incognita* are needed.

Biological control with microbial antagonists has received a great deal of attention as a promising measure to control different plant diseases [6, 7]. Many antagonistic microorganisms including *Trichoderma* spp., *Streptomyces* spp., *Pseudomonas* spp., *Bacillus* spp. have been screened and widely exploited to control a wide range of plant pathogens [8–11]. Some microorganisms have been identified as biological control agents against *M. incognita* such as *Pasteuria penetrans*, *B. subtilis*, *T. harzianum* and *T. viride*, *Pochonia chlamydosporia* and *Purpureocillium lilacinum* [12–16]. *Trichoderma* genus is one of the most frequently studied groups of fungi used as biological control agents. Its species are often very fast growing, rapidly colonize substrates and effectively control different diseases by using a variety of mechanisms [17, 18]. At present, there are no reports about using *T. citrinoviride* as a biological control agent against RKN, *M. incognita*.

The objective of this study was to isolate effective fungal strains against *M. incognita*, evaluate the biological control activity under in vitro and in vivo, phylogenetically identify them based on morphology and sequence analysis and their effects on the growth of tomato plants in pot and field experiments.

## Results

### Screening of antagonistic fungi

**Fig. 1:**
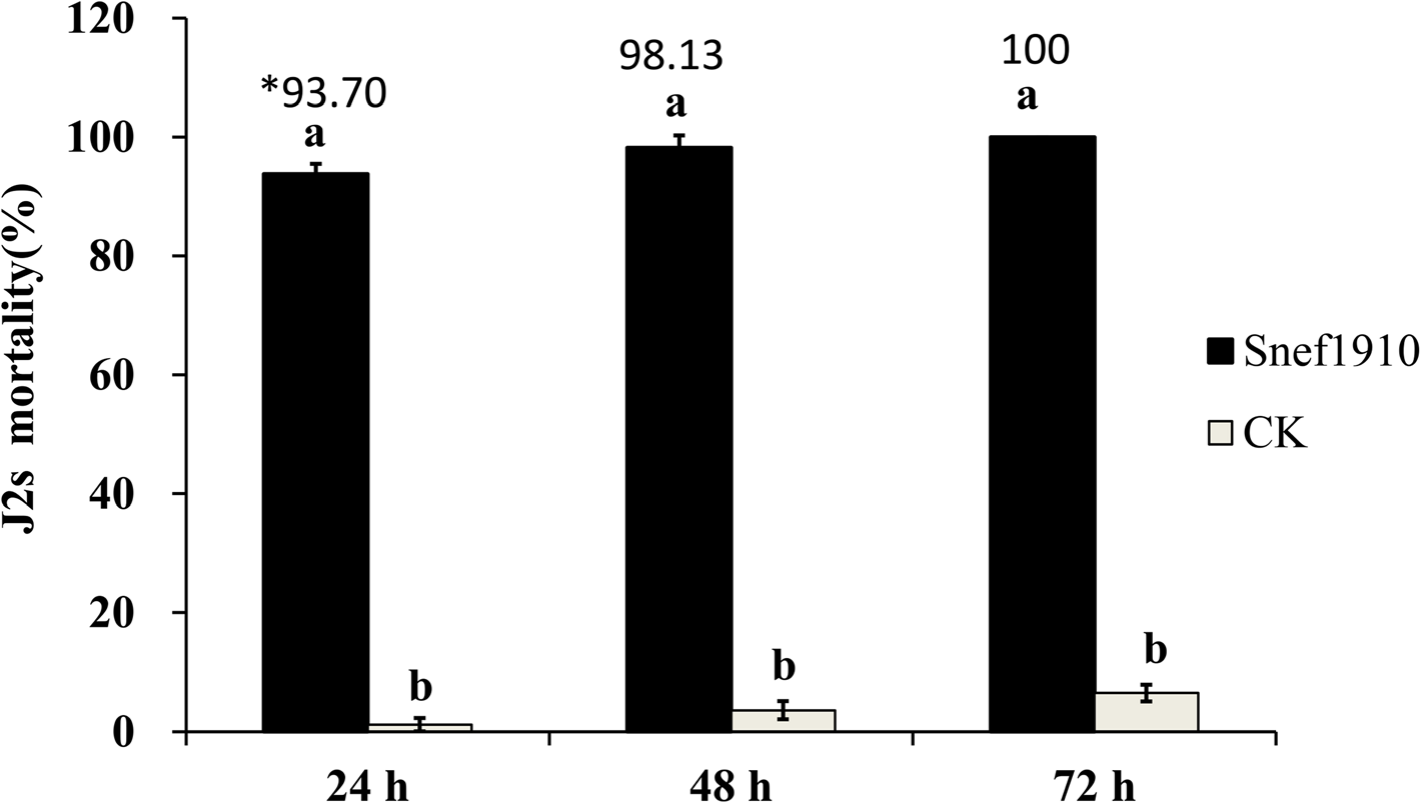
The effect of strain Snef1910 on second stage juvenile (J2s) mortality of *M. incognita* in vitro. The J2s suspension mixed with PDB medium alone was used as control (CK). Error bars represent standard deviation. ^∗^The data on the columns represent the corrected J2s mortality. J2s mortality (%) = the number of dead J2s/total number of J2s × 100. Corrected J2s mortality (%) = (J2s mortality in the treatment – J2s mortality in the control)/(100 – J2s mortality in the control) × 100

In total, 890 fungi isolates were obtained from rhizosphere soil of different crops and screened for the potential nematicidal action against *M. incognita* in vitro (Table S1). Among these isolates, strain Snef1910 (CGMCC Accession No.13569; China General Microbiology Culture Collection Center) showed the strongest nematicidal activity against second stage juveniles (J2s) of *M. incognita.* The percentages of J2s mortality of strain Snef1910 were 93.79, 98.20, and 100% at 24 h, 48 h, and 72 h, respectively (Fig. 1). Moreover, strain Snef1910 showed significant antagonistic activity in vitro towards other pathogens that caused plant diseases in wheat, cotton, melon and other plants (Table 1).

**Table 1:** Inhibitory activity of strain Snef1910 against various fungal plant pathogens in vitro

Target fungal pathogens	the percentage of growth inhibition
*Fusarium graminearum*	60.76%
*Fusarium oxysporum*	49.28%
*Fusarium monihforme*	21.73%
*Fusarium roseum*	25.20%
*Gaeumannomyces graminis*	62.31%
*Rizoctonia cerealis*	80.38%
*Verticillium alboatrum*	54.87%

### Evaluation of ovicidal efficacy in vitro

**Fig. 2:**
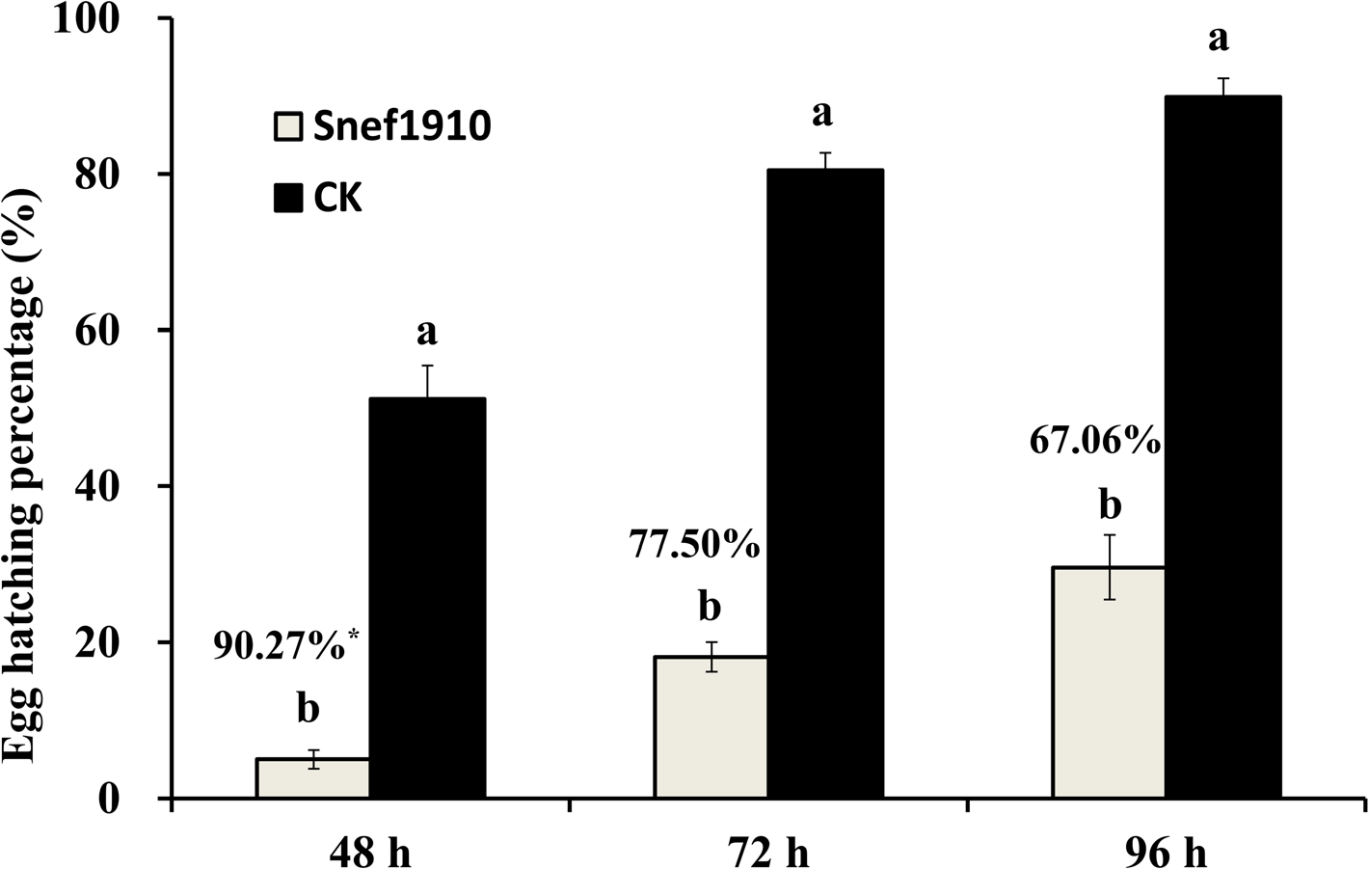
The egg inhibition of *M. incognita* exposed to strain Snef1910 culture filtrate in vitro. CK denotes for the PDB medium. ^∗^The data on the columns represent the hatch inhibition percentage. Egg hatching percentage (%) = the number of hatched eggs/total number of eggs × 100. Hatch inhibition percentage (%) = (the number of hatched eggs in the control – the number of hatched eggs in the fungus-treated group)/the number of hatched eggs in the control × 100

To further investigate the nematicidal activity of strain Snef1910, the ovicidal efficacy against *M. incognita* was performed. The egg hatching percentage of *M. incognita* treated with PDB medium (control) was 51.17% at 48 h, while in treatment with strain Snef1910 culture filtrate it was 4.98% (Fig. 2). The hatch inhibition percentage of strain Snef1910 was up to 90.27% (Fig. 2). Similar results were obtained at 72 h and 96 h (Fig. 2).

### Identification of strain Snef1910

**Fig. 3:**
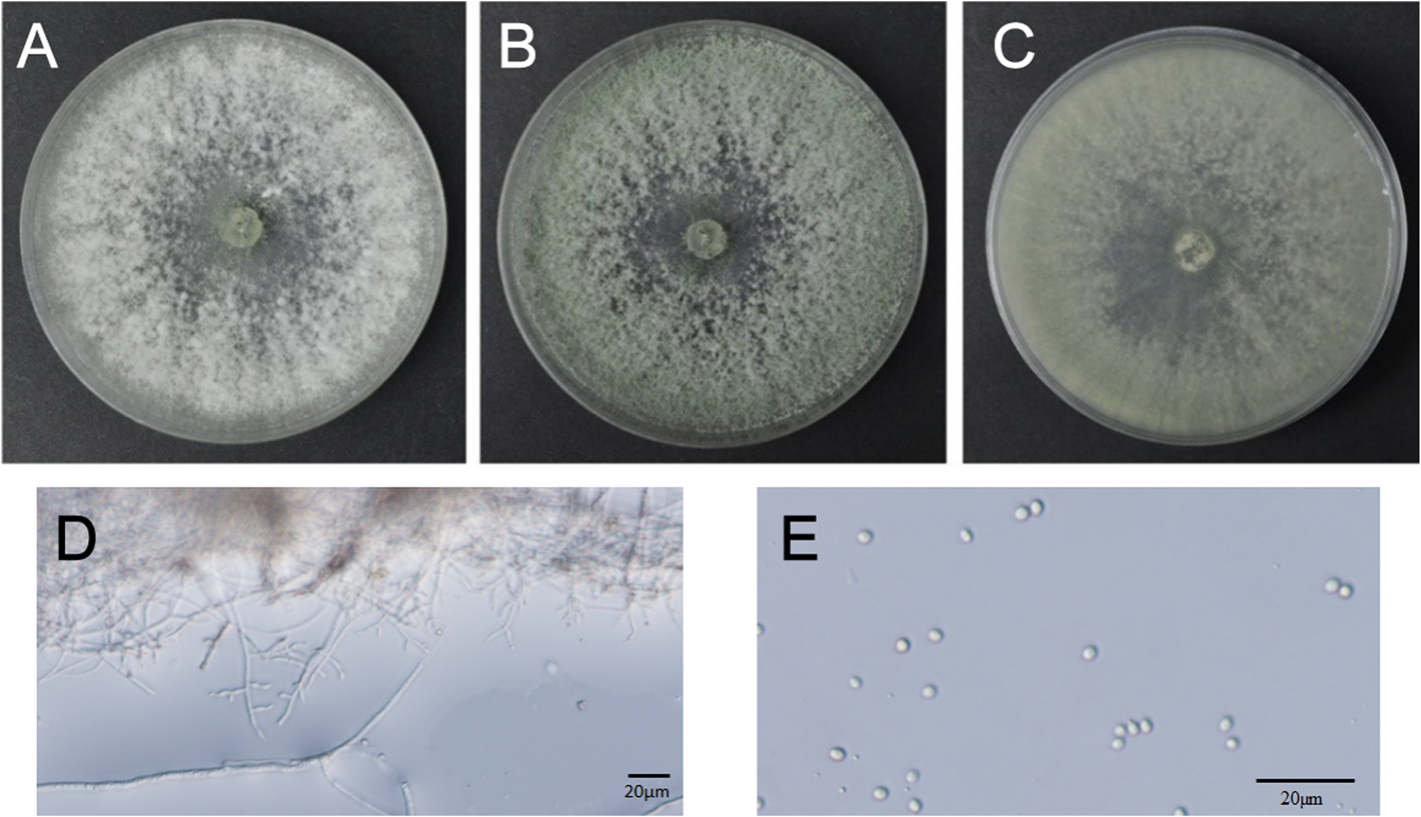
Colony characteristics and conidiophores and conidia of strain Snef1910. **a-c** Colony appearance of strain Snef1910 grown on PDA at 25 °C for 2–7 days. **d** Microscopic image showing conidiophores structures and branching pattern of strain Snef1910. **e** Image of conidia of strain Snef1910

To identify strain Snef1910, morphological observation was performed. The strain Snef1910 grew rapidly on PDA medium forming a white, 60-mm-diameter colony at 25 °C under dark for 2 days. Then, the colony changed to greyish green or dark green and formed a wide conidial zone at the edge of the colony at 7 days (Fig. 3a-c). The conidiophores were erect showing a long axis of the structure and fertile to the top. The cylindrical or spindle-shaped bottle stems were solitary born on main branch and spirally arranged on top of lateral branches (Fig. 3d). The conidia were yellowish green to dark green, globose with smooth walls (Fig. 3e). No distinct coconut-like odor was detected. Based on the above microscopic observations, strain Snef1910 was tentatively identified as *Trichoderma*.

**Fig. 4:**
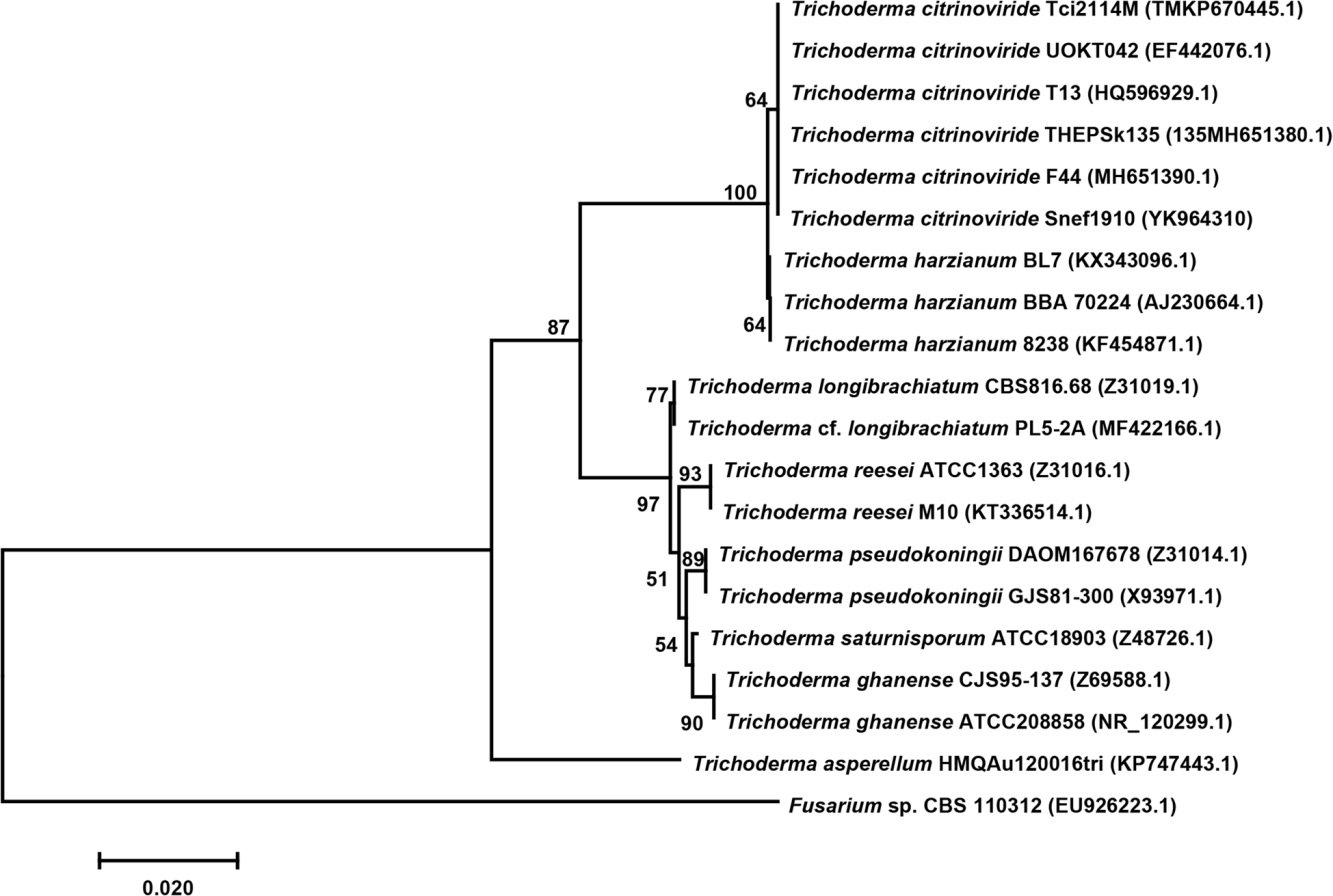
Phylogenetic tree of strain Snef1910 based on the partial nucleotide sequences of 5.8S-ITS. A neighbor-joining phylogenetic tree of strain Snef1910 was constructed using MEGA 4.0. The percentage numbers at the nodes indicate the levels of bootstrap support based on a neighbor-joining analysis of 1000 resampled datasets; only values greater than 50% are provided. The scale bar indicates 0.020 nucleotide substitutions per nucleotide position

To further identify strain Snef1910, a phylogenetic analysis of its 5.8S-ITS sequences was performed. The phylogenetic tree showed that the 5.8S-ITS sequence (GenBank accession number: YK964310) of strain Snef1910 was clustered with *T. citrinoviride* (Fig. 4). These results demonstrated that strain Snef1910 was identified as *T. citrinoviride*.

### Biocontrol of root-knot nematode in the pot experiment

In order to investigate the potential biological control activity of *T. citrinoviride* Snef1910 against *M. incognita* in vivo, tomato seedlings were treated with a fermentation broth of *T. citrinoviride* and inoculated with J2s of *M. incognita*. The number of root galls on tomato seedlings treated with PDB culture medium (PDB culture medium-inoculated) was 47.35, thirty days post-inoculation of *M. incognita*, while the number of root galls on tomato seedlings treated with fermentation broth of *T. citrinoviride* Snef1910 and untreated were 16.05 and 48.15, respectively (Table 2). The number of root galls on tomato seedlings treated with *T. citrinoviride* Snef1910 was reduced by 66.10% compared with that of the PDB culture medium-inoculated (Table 2). *T. citrinoviride* Snef1910 caused significant decreases in the number of egg masses and J2s on tomato seedling root, which were 80.63 and 69.87% reduced, respectively, compared with those in the control (PDB culture medium-inoculated) (Table 2). Furthermore, thirty days after *M. incognita* inoculation, the number of J2s per 100 mL soil of *T. citrinoviride* Snef1910-treated seedlings was significantly smaller than that of the control (PDB culture medium-inoculated), with mortality reaching 77.28% (Table 2). Thirty days after treatment, *T. citrinoviride* Snef1910-treated plants exhibited increases in shoot length, root length, root fresh weight, and root dry weight by 15.61, 23.32, 35.08, and 33.33%, respectively, compared with untreated plants (Table 3).

**Table 2:** The biological control activity of *T. citrinoviride* Snef1910 against *M. incognita* in the pot experiment with tomato plants

Treatments	Root galls/ g root	Egg masses/ g root	Juveniles/ g root	J2s/100 ml soil
untreated-inoculated	48.15 ± 5.40^a^	43.85 ± 2.90^a^	65.80 ± 3.40^a^	308.30 ± 22.33^a^
PDB culture medium-inoculated	47.35 ± 3.72^a^	42.60 ± 2.88^a^	67.20 ± 3.89^a^	297.10 ± 15.83^a^
Snef1910-inoculated	16.05 ± 2.27^b^	8.25 ± 1.62^b^	20.25 ± 2.15^b^	67.50 ± 6.35^b^

The biological control activity of *T. citrinoviride* Snef1910 against *M. incognita* in the pot experiment was determined, 30 days post-inoculation. The data are the averages ± standard error from ten replicates. Different letters represent a significant difference at *P* ≤ 0.05

**Table 3:** The effects of *T. citrinoviride* Snef1910 on the growth parameters of tomato plants in pot experiments

Treatments	Shoot length (cm)	Root length (cm)	Root fresh weight (g)	Root dry weight (g)
untreated-uninoculated	42.72 ± 2.83^b^	15.13 ± 1.36^b^	7.57 ± 0.82^ab^	0.80 ± 0.08^b^
untreated-inoculated	35.40 ± 3.87^c^	10.45 ± 0.72^c^	4.46 ± 0.66^c^	0.52 ± 0.06^c^
PDB culture medium-inoculated	35.89 ± 3.43^c^	11.08 ± 1.03^c^	4.57 ± 0.75^c^	0.52 ± 0.07^c^
Snef1910-uninoculated	46.98 ± 1.76^a^	16.90 ± 1.08^a^	8.28 ± 0.92^a^	0.95 ± 0.08^a^
Snef1910-inoculated	42.53 ± 3.10^b^	14.45 ± 2.12^b^	7.04 ± 0.51^b^	0.78 ± 0.08^b^

The effects of *T. citrinoviride* Snef1910 on the growth parameters of tomato plants in the pot experiment was determined, 30 days post-inoculation. The data are the averages ± standard error from ten replicates. Different letters represent a significant difference at *P* ≤ 0.05

### Biocontrol of root-knot nematode in the field experiment

**Fig. 5:**
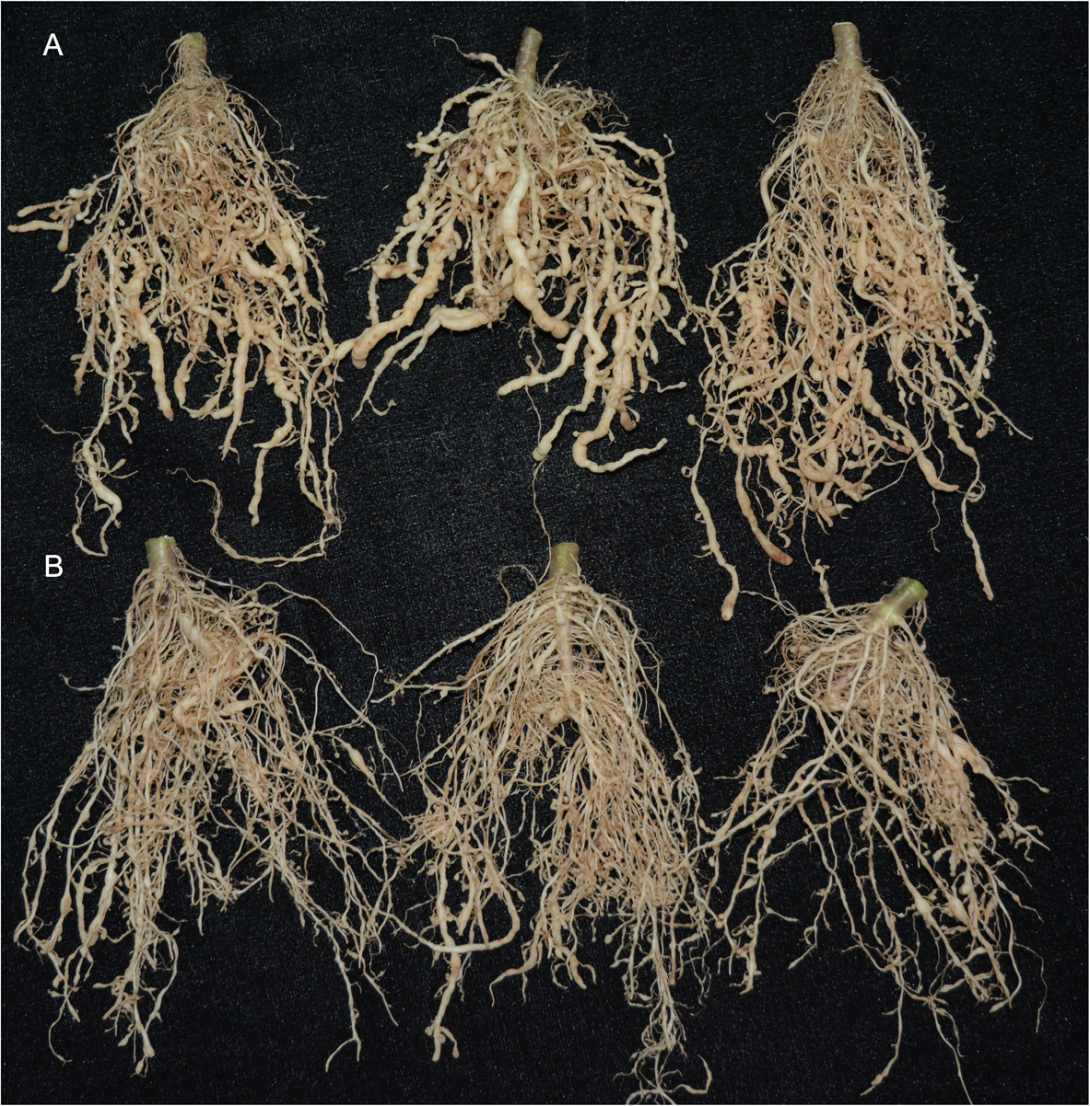
Biocontrol of *T. citrinoviride* Snef1910 against *M. incognita* in the field experiment from November to December in 2019. **a** The *M. incognita* infected tomato plant roots, **b** the biocontrol effect of *T. citrinoviride* Snef1910 on tomato plant roots. Root gall indices were rated using a scale of 0–5, where 0, no gall; 1,15% or less roots with galls; 2–4, 16–25%; 26–50%; 51–75% roots with galls, respectively; and 5,> 76% roots with galls. Gall index = ∑the number of diseased plants in each grade × grade/(total number of plants investigated × the highest grade) × 100%. Biocontrol efficacy (%) = (gall index in the control − gall index in the Snef1910-treated group)/ gall index in the control × 100

The field experiments showed similar results to the greenhouse pot experiments. At 30 days after transplantation, the gall index of seedlings treated with *T. citrinoviride* Snef1910 were 42.67 and 37.78 while those treated with PDB (control) were 86.67 and 80.00 (Table 4) which indicated a noticeable reduction of RKN during a two-growth seasons of field experiment. Furthermore, the biocontrol efficacy of *T. citrinoviride* Snef1910 in controlling *M. incognita* reached up to 50.77 and 52.77% in the field experiments (Table 4; Fig. 5). These results indicated that *T. citrinoviride* Snef1910 is a biological control agent for efficiently controlling RKN. Shoot length, root length, fresh weight of root were also measured in the field experiments. The growth-promoting results in field experiments were similar to the greenhouse pot experiments. The shoot length and root length in *T. citrinoviride* Snef1910 treated plants increased by 13.48 and 17.98%, respectively, in comparison with the control plants (Table 5), which suggested that *T. citrinoviride* Snef1910 promotes the growth of tomato. In contrast, *T. citrinoviride* Snef1910 showed a 35.62% reduction in the fresh weight of root, thirty days after transplantation (Table 5). Similar results were obtained in the second field experiment (November to December in 2019) (Table 5). These results suggested that *T. citrinoviride* Snef1910 has the potential in increasing tomato plant growth.

**Table 4:** The biocontrol effect of *T. citrinoviride* Snef1910 against *M. incognita* in the field experiment

Treatments	August to September in 2019		November to December in 2019	
	Gall index	Biocontrol efficacy (%)	Gall index	Biocontrol efficacy (%)
Snef1910	42.67 ± 2.31^a^	50.77	37.78 ± 3.85^a^	52.77
Control	86.67 ± 2.31^b^	–	80.00 ± 6.67^b^	–

The biocontrol effect of *T. citrinoviride* Snef1910 against root-knot nematode disease caused by *M. incognita* in the field experiments was determined, 30 days after transplantation. The data are the averages ± standard error from ten replicates. Different letters represent a significant difference at *P* ≤ 0.05

**Table 5:** The effect of *T. citrinoviride* Snef1910 on the growth parameters of tomato plants infected with *M. incognita* in the field experiments

Treatments	August to September in 2019			November to December in 2019		
	Shoot length (cm)	Root length (cm)	Root fresh weight (g)	Shoot length (cm)	Root length (cm)	Root fresh weight (g)
Snef1910	99.40 ± 1.14^a^	17.80 ± 0.84^a^	47.55 ± 5.28^a^	91.20 ± 1.30^a^	16.00 ± 0.71^a^	8.35 ± 1.38^a^
Control	86.00 ± 2.24^b^	14.60 ± 1.67^b^	73.86 ± 4.32^b^	73.20 ± 4.09^b^	14.20 ± 1.48^b^	12.52 ± 2.82^b^

The effects of *T. citrinoviride* Snef1910 on the growth parameters of tomato plants in the pot experiment was determined, 30 days after transplantation. The data are the averages ± standard error from ten replicates. Different letters represent a significant difference at *P* ≤ 0.05

## Discussion

Researches on the use of antagonistic microorganisms to suppress plant diseases are receiving increasingly attention [19]. In recent years, *Trichoderma* spp. has been used as biocontrol agents to control different plant pathogens [20, 21]. Specifically, previous studies have reported that *T. viride* MTCC No. 167, *T. asperellum* T-12, and *T. harzianum* T-78 were potential biological agents against *M. incognita* [22–25]. Furthermore, a few other microorganisms such as *S. rubrogriseus*, *B. subtilis*, *P. putida and P. fluorescens* have been employed in controlling *M. incognita* [13, 26]. However, the microorganism sources used for the control of *M. incognita* are limited and need further explored. In this study, *T. citrinoviride* Snef1910 strain was screened from 890 fungal isolates and showed high larvicidal and ovicidal activity against *M. incognita* in vitro. Furthermore, *T. citrinoviride* Snef1910 strain significantly reduced the galls and J2s of nematodes, and promoted the growth of tomato plant in the pot and field trials. This study is the first report on the use of a *T. citrinoviride* strain as a potential biological control agent to control RKN disease. The results of our study provide an alternative and promising biological control agent and practical strategies for controlling RKN, *M. incognita*.

*Trichoderma* species have been reported as biological control agents in controlling RKN disease [23–25]. For example, nematode galling caused by *M. incognita* on tomato showed reductions of up to 30.8% under the soil treatments with *T. harzianum* and *T. viride* [27]. Meanwhile, *T. harzianum* can reduced galling caused by another RKN *M. javanica* on tomato plants [28]. In addition, the biocontrol efficacy of another species *T. longibrachiatum* in controlling *M. incognita* was indicated from 30 to 78% for gall index on cucumber [29]. In the present study, the damage of RKN can reduce over 50%, which suggested that *T. citrinoviride* Snef1910 has more biocontrol efficiency and potential application to control RKN *M. incognita* on tomato.

Multiple action modes of *Trichoderma* species were reported to contribute to the biological control, including nutrient and space competition, antibiosis, mycoparasitism, and induced systemic resistance of plants [30–33]. Particularly, the antimicrobial compounds produced by *Trichoderma* species and their roles in controlling plant pathogens have been characterized [34–36]. For example, acetic acid isolated from culture filtrates of *T. longibrachiatum* showed nematicidal activity against *Meloidogyne* spp. [37]. Many biocontrol activators obtained from *Trichoderma* spp. have been proven to be nematicidal compounds such as trichodermin and trypsin-like protease [38–41]. Our results indicated that cultural filtrates of *T. citrinoviride* Snef1910 strain showed high ovicidal and larvicidal activities (Fig. 1, 2). Further research will be conducted to identify and characterize the nematicidal compounds of *T. citrinoviride* Snef1910 against *M. incognita*.

The effective ways of biocontrol products and microorganism application should be applied according to the characteristics of the biocontrol agents and the routine practices of agricultural producers [42]. For example, the soil treatment with the culture broth of *B. subtilis* isolate B10 was highly significant in decreasing number of galls and egg masses of *M. incognita* with reduction percentage of 81.1 and 89.5%, respectively [43]. Application of the fermentation broth of five bacterial strains by coating tomato seeds showed high biocontrol efficacy against *M. incognita* [26]. Moreover, soil treatment with the biocontrol agents *T. harzianum* and *T. viride* improved the efficiency of nematode control by reductions of up to 30.8% in nematode galling on tomato [27, 44]. Na et al. [5] reported that drenching of the broth containing spores was the appropriate application method for *S. rubrogriseus* HDZ-9-47 to control *M. incognita,* which reduced the root knot index and J2s density by 51.1 and 80.7%, respectively. In this study, *T. citrinoviride* Snef1910 significantly reduced the galls and nematodes in the pot and field trials through application the fermentation broth (Tables 2, 4). Therefore, drenching the fermentation broth is one of the application approaches for *T. citrinoviride* Snef1910 to control RKN. Meanwhile, other application such as seed coating of *T. citrinoviride* Snef1910 will be expected to study in the future.

Moreover, the plant growth promotion is well characterized in *Trichoderma* species. For example, *T. harzianum* T969 increased the shoot height (58.70%), shoot diameter (58.03%), root fresh weight (78.92%) and dry weight (93.07%) of tomato seedlings [45]. The mechanisms of *Trichoderma* in promoting plant growth also involve the production of auxin-like compounds, improving availability of nutrients, affecting root system, and inducing of systemic resistance phenomenon [46–49]. For example, the releasing an auxin-like phytohormone of *T. harzianum* SQR-T037 significantly promoted tomato seedling growth by up to 2.5-fold dry weight [46]. In this study, *T. citrinoviride* Snef1910 resulted in increased plant growth of tomato in the pot and field experiments (Tables 3, 5). More work is also planned to explore the growth promotion effects and mechanisms of *T. citrinoviride* Snef1910 in tomato and even other plants.

## Conclusions

In conclusion, this study showed that *T. citrinoviride* Snef1910 was screened from 890 fungi isolates and efficiently controlled RKN disease caused by *M. incognita*, which played control efficacy of more than 50% and increased egg hatching inhibition percentages and reduced root galls, egg masses and J2s on tomato. Moreover, *T. citrinoviride* Snef1910 showed the plant growth promotion of shoot and root length of tomato. This is the first report on the use of a *T. citrinoviride* strain as a potential biological control source to control RKN disease caused by *M. incognita*. This study provides a new biological control agent and potentially practical strategies for sustainable management of RKN.

## Methods

### Isolation of fungal strains

Fungal strains used in this study were originally isolated from rhizosphere soil collected from health plants including tomatoes, cucumbers, soybean, eggplant, peanut, and corn in different locations in northeast China. For fungal isolation, the soil serial dilution plate method was used [50]. In brief, a 1 g of rhizosphere soil samples was added into 9 mL of sterilized water and then mixed to obtain soil suspension. The soil suspension was serially diluted to the appropriate concentration (10^− 2^ g/mL and 10^− 3^ g/mL) and plated on Potato Dextrose Agar (PDA) plates. The plates were then incubated at 28 °C for 5 days. Individual fungal colonies were isolated and purified.

### Preparation of nematode inoculum

The root-knot nematode *M. incognita*, which sampled from the invasive tomato field located in Tieling County (123.92E, 42.18 N), Liaoning Province, China, was maintained on tomato plants in the greenhouse of Nematology Institute of Northern, Shenyang Agricultural University, China. Eggs were separated from masses that collected from the roots of tomato plants according to the method of Martinuz et al. [51]. The J2s were obtained by incubating the eggs in sterile water at 25 °C for 5 days and collected every 24 h. Then, the J2s were diluted to the appropriate concentration and used for further study.

### Screening of fungal isolates against *M. incognita* in vitro

The J2s of *M. incognita* were used to detect the nematicidal activities of fungal isolates. The fermentation broth of fungal strains was prepared in Potato Dextrose Broth (PDB) medium in a shaker at 150 rpm for 5 days at 28 °C. Cell-free supernatant was obtained after centrifugation at 4500×g for 15 min and filtered using the filter (φ = 0.45 μm). A 50 μL suspension containing 50 J2s was added into each petri dish with 950 μL of the prepared fungal cell-free supernatant. The J2s suspension mixed with PDB medium alone was used as control. Five dishes were used for each treatment. All the dishes were incubated at 25 °C for 72 h. The number of dead and alive J2s was examined using the stereoscopic microscope at 24 h, 48 h, and 72 h after treatment. A nematode that malformed, immobile or motionless even probed with a fine needle was deemed dead [52]. The J2s mortality and corrected J2s mortality were calculated as following:

$$ \mathrm{J}2\mathrm{s}\ \mathrm{mortality}\ \left(\%\right)=\mathrm{the}\ \mathrm{number}\ \mathrm{of}\ \mathrm{dead}\ \mathrm{J}2\mathrm{s}/\mathrm{total}\ \mathrm{number}\ \mathrm{of}\ \mathrm{J}2\mathrm{s}\kern0.5em \times 100. $$

$$ \mathrm{Corrected}\ \mathrm{J}2\mathrm{s}\ \mathrm{mortality}\ \left(\%\right)=\left(\mathrm{J}2\mathrm{s}\ \mathrm{mortality}\ \mathrm{in}\ \mathrm{the}\ \mathrm{treatment}\hbox{-} \mathrm{J}2\mathrm{s}\kern0.2em \mathrm{mortality}\ \mathrm{in}\ \mathrm{the}\ \mathrm{control}\right)/\left(100\hbox{-} \mathrm{J}2\mathrm{s}\ \mathrm{mortality}\ \mathrm{in}\ \mathrm{the}\ \mathrm{control}\right)\times 100 $$

The strains that exhibited the strongest nematicidal activity against J2s of *M. incognita* were chosen for the future study. The effects of selected strains on J2s mortality of *M. incognita* were conducted as described above and repeated three times.

### In vitro antagonism test

The dual culture technique was used to detect the antagonistic activities of strain Snef1910 against fungal plant pathogens (*Fusarium graminearum*, *F. oxysporum*, *F. moniliforme*, *F. roseum*, *Gaeumannomyces graminis*, *Rhizoctonia cerealis*, *Verticillium alboatrum*) [53]. In brief, the pathogen discs (5 mm diameter) were placed on one side of PDA plates (90 mm) around the center at a distance of 3 cm. The plugs of strain Snef1910 was placed on the opposite side and around the fungal inocula at a distance of 6 cm. PDA plates inoculated with the pathogen alone were used as a control. The plates were incubated at 28 °C for 5 days. The antagonistic activity of strain Snef1910 was assessed by measuring the colony diameters. The percentage of growth inhibition was calculated using the formula R = (a–b)/a × 100, where R, a, and b is the percentage of growth inhibition, the mycelial radial growth of the pathogen in the control and in the presence of the antagonist, respectively. The values were recorded as the means of three replicates, and the experiments were repeated three times. The 7 plant pathogens tested in this study were kindly provided by the College of Plant Protection, China Agricultural University, China.

### Testing the ovicidal efficacy of strain Snef1910 against *M. incognita* in vitro

The ovicidal efficacy of strain Snef1910 against *M. incognita* was performed as previously described [26]. In short, 200 eggs were carefully added to 500 μL of strain Snef1910 metabolites in each well of 96-well microtiter plates. Eggs transferred into PDB medium alone were used as controls. The plates were incubated at 28 °C for 96 h. In order to stop further hatching, 500 μL of Lugol’s iodine solution was added to each well [25]. The numbers of the unhatched eggs were counted under microscope [5], which were used to calculate the hatching percentage, hatch inhibition and corrected egg inhibition by the following formula:

$$ \mathrm{Egg}\ \mathrm{hatching}\ \mathrm{percentage}\ \left(\%\right)=\mathrm{the}\ \mathrm{number}\ \mathrm{of}\ \mathrm{hatched}\ \mathrm{eggs}/\mathrm{total}\ \mathrm{number}\ \mathrm{of}\ \mathrm{eggs}\times 100. $$

$$ \mathrm{Hatch}\ \mathrm{in}\mathrm{hibition}\ \left(\%\right)=\left(\mathrm{the}\ \mathrm{number}\ \mathrm{of}\ \mathrm{hatched}\ \mathrm{eggs}\ \mathrm{in}\ \mathrm{the}\ \mathrm{control}-\mathrm{the}\ \mathrm{number}\ \mathrm{of}\ \mathrm{hatched}\ \mathrm{eggs}\ \mathrm{in}\ \mathrm{the}\ \mathrm{fungus}-\mathrm{treated}\ \mathrm{group}\right)/\mathrm{the}\ \mathrm{number}\ \mathrm{of}\ \mathrm{hatched}\ \mathrm{eggs}\ \mathrm{in}\ \mathrm{the}\ \mathrm{control}\times 100. $$

$$ \mathrm{Corrected}\ \mathrm{egg}\ \mathrm{inhibition}\ \left(\%\right)=\left(\mathrm{The}\ \mathrm{hatch}\ \mathrm{in}\mathrm{hibition}\ \mathrm{in}\ \mathrm{the}\ \mathrm{treatment}-\mathrm{the}\ \mathrm{hatch}\mathrm{ed}\ \mathrm{in}\mathrm{hibition}\ \mathrm{in}\ \mathrm{control}\right)/\left(100-\mathrm{hatch}\ \mathrm{in}\mathrm{hibition}\ \mathrm{in}\ \mathrm{the}\ \mathrm{control}\right) $$

Three wells were used for each replicate, and the values were recorded as the means of three replicates for each treatment. Ovicidal experiments were repeated three times.

### Identification of strain Snef1910

To identify strain Snef1910, the growth pattern and microscopic observation of the morphology of conidia and conidiophores were performed. Single spore of strain Snef1910 was grown on the PDA plate at 25 °C for 5 days. Then, a 5-mm-diameter fungal plug was placed in the center of PDA plates (90 mm) at 25 °C in the dark for 7 days. The color, smell, growth, and shape of the colony and conidiophores branching pattern and conidia were examined [54, 55].

Afterwards, strain Snef1910 was further identified through a phylogenetic analysis of its 5.8S-ITS region sequence. Genomic DNA of Snef1910 strain was extracted using a N96 Plant Genomic DNA Kit (Tiangen Biotech Co., Ltd., Beijing, China) according to the manufacturer’s instructions. The fragment of 5.8S-ITS region was amplified with primer pair ITS1 and ITS4 [56]. The PCR product was ligated into the pMD19-T vector (Takara Co., Ltd., Dalian, China), and the resulting recombinant plasmid was sent to Shanghai Sangon Biotechnology Co., Ltd. (Shanghai, China) for sequencing. The phylogenetic tree of strain Snef1910 based on the sequence of 5.8S-ITS region was constructed using the neighbor-joining method in MEGA 4.0 software [57]. The topology of the phylogenetic tree was evaluated using1000 bootstrap resampling replicates.

### Biocontrol of root-knot nematode in the pot experiments

The tomato (*Solanum lycopersicum* cultivar L-402 susceptible to *M. incognita*) was used to evaluate the biocontrol potentials of strain Snef1910. Tomato seeds were surface sterilized with 1% (v/v) sodium hypochlorite solution for 5 min, followed by 3 times washings with sterile distilled water [58]. Three seeds were sown in plastic pots (5 × 10 holes) filled with 42 g of soil, vermiculite and sand at a ratio of 2:1:1. The sand and soils were autoclaved at 165 °C for 120 min before planting. During the entire course of the experiment, the plants were watered two times and fertilized Hoagland solution once a week [24]. After attaining 2-leaf stage, the tomato seedlings were transplanted into 13 cm × 12 cm plastic pots containing 600 g of autoclaved soil, sand and vermiculite at a ratio of 2:1:1 (one tomato seedling per pot). The pots were placed in a greenhouse under the following conditions: 28 ± 2 °C, 60% humidity, and 16 h of light alternating with 8 h of darkness.

The fermentation broth (2.15 × 10^6^ spores/mL) of strain Snef1910 was prepared by the method described above. After transplanting for 2 days, each pot was treated with 2 mL fermentation broth of strain Snef1910. At the same time, each seedling was inoculated with 5 mL of *M. incognita* suspension containing 2000 motile J2s, and named Snef1910-inoculated. In addition, the following four treatments were maintained as controls: untreated + uninoculated control (untreated-uninoculated), untreated-uninoculated + inoculated *M. incognita* (untreated-inoculated), 2 mL of fermentation broth of strain Snef1910-uninoculated (Snef1910-uninoculated), and 2 mL PDB culture medium-inoculated (PDB culture medium-inoculated). The fermentation broth of strain Snef1910 and PDB culture medium filtrate in treatments of PDB culture medium-inoculated, Snef1910-uninoculated, and Snef1910-inoculated were suspended in 100 mL of sterile distilled water and applied by drenching roots. Each treatment included 10 replicates, and each replicate included one tomato plant. The experimental design for pot assay was performed a randomized complete block design.

Thirty days after inoculation, the tomato plants with their rhizosphere soil samples were collected. To evaluate the biocontrol potentials of strain Snef1910, shoot length, root length, fresh and dry weights of root, the number of root galls, J2s and egg masses per 1 g root, and J2s per 100 mL soil were counted as previously described [26, 59]. The experiments were repeated three times.

### Biocontrol of root-knot nematode in the field experiment

The field experiment was carried out in a field naturally and severely infested with *M. incognita*, located in Tieling County, Liaoning Province, China (123.92E, 42.18 N) in two growing seasons (from August to September and November to December) in 2019 with the temperature ranged from 15 °C to 35 °C. The soil in protected field was determined as a brown soil with the properties of pH 5.56 ± 0.08, organic matter 70.76 g /kg, and total nitrogen, available potassium, and available phosphorus of 4.56, 285.95, and 81.68 mg/kg, respectively. The four-week-old tomato seedlings without infestation with *M. incognita* were transplanted into the field. The 196 mL sterile distilled water was added into 4 mL fermentation broth (10^6^ spores/mL) of strain Snef1910 or 4 mL PDB culture medium to obtain the desired concentrations and poured into the planting hole during plant transplantation. The experimental plots were 6 m long, 5.5 m wide and separated by 0.5 m (6 plant rows) and contained 21 transplanted seedlings per row. A randomized complete block design was adopted in this experiment, and each treatment consisted of three replications. The protected fields were irrigated and fertilized followed by farming practice.

Fifteen plants and rhizosphere soil samples were randomly selected and collected form each treatment, 30 days after transplantation. The shoot length, root length, and fresh weight of root were measured as described above. Root gall indices were rated using a scale of 0–5, where 0, no gall; 1,15% or less roots with galls; 2–4, 16–25%; 26–50%; 51–75% roots with galls, respectively; and 5,> 76% roots with galls [60]. The root gall index and biocontrol efficacy were calculated as follow:

$$ \mathrm{Gall}\ \mathrm{in}\mathrm{dex}=\sum \mathrm{the}\ \mathrm{number}\ \mathrm{of}\ \mathrm{diseased}\ \mathrm{plants}\ \mathrm{in}\ \mathrm{each}\ \mathrm{grade}\times \mathrm{grade}/\left(\mathrm{total}\ \mathrm{number}\ \mathrm{of}\ \mathrm{plants}\ \mathrm{in}\mathrm{vestigated}\times \mathrm{the}\ \mathrm{highest}\ \mathrm{grade}\right)\times 100\%. $$

$$ \mathrm{Biocontrol}\ \mathrm{efficacy}\ \left(\%\right)=\left(\mathrm{gall}\ \mathrm{in}\mathrm{dex}\ \mathrm{in}\ \mathrm{the}\ \mathrm{control}-\mathrm{gall}\ \mathrm{in}\mathrm{dex}\ \mathrm{in}\ \mathrm{the}\ \mathrm{Snef}1910-\mathrm{treated}\ \mathrm{group}\right)/\mathrm{gall}\ \mathrm{in}\mathrm{dex}\ \mathrm{in}\ \mathrm{the}\ \mathrm{control}\times 100. $$

### Statistical analysis

Data were statistically analyzed using SPSS software 20.0. Duncan’s one-way analysis of variance was used to determine the significant differences.

## Supplementary information

**Additional file 1: Table S1**. The effect of fermentation broth of 890 fungi strains on second stage juvenile (J2s) mortality of *M. incognita* in vitro at 24 h.

## Data Availability

The datasets used and analysed during the current study are available from the corresponding author on reasonable request. All data generated or analysed during this study are included in this article.

## Abbreviations

J2s: Second stage juveniles
PCR: Polymerase Chain Reaction
PDA: Potato Dextrose Agar
PDB: Potato Dextrose Broth
RKN: Root-knot nematode
