# Supplementary material for: Isolation and effect of Trichoderma citrinoviride Snef1910 for the biological control of root-knot nematode, Meloidogyne incognita
Source: BMC Microbiol. 2020 Oct 2;20:299. doi: 10.1186/s12866-020-01984-4 (PMC7531111; doi:10.1186/s12866-020-01984-4)
Supplement: Supplementary file 1 — Additional file 1: Table S1. The effect of fermentation broth of 890 fungi strains on second stage juvenile (J2s) mortality of M. incognita in vitro at 24 h. [file 12866_2020_1984_MOESM1_ESM.docx]

**Table S1**

The effect of fermentation broth of 890 fungi strains on second stage juvenile (J2s) mortality of *M. incognita in vitro* at 24 h.

| Corrected J2s mortality (%) | strains |
| --- | --- |
| 0-25.00 | Snef458, Snef1956, Snef219, Snef2658, Snef45, Snef1114, Snef1190, Snef1039, Snef1008, Snef325, Snef958, Snef241, Snef957, Snef2489, Snef1181, Snef1602, Snef2424, Snef34, Snef1790, Snef386, Snef213, Snef682, Snef35, Snef690, Snef2304, Snef2531, Snef202, Snef1099, Snef2470, Snef2484, Snef2554, Snef262, Snef2592, Snef2580, Snef842, Snef1789, Snef382, Snef1288, Snef319, Snef2540, Snef1952, Snef1165, Snef1774, Snef2384, Snef2375, Snef1269, Snef2291, Snef1151, Snef1198, Snef1473, Snef2374, Snef344, Snef2478, Snef936, Snef1268, Snef22, Snef105, Snef1380, Snef291, Snef1321, Snef1328, Snef1645, Snef882, Snef2321, Snef553, Snef1287, Snef1442, Snef215, Snef2299, Snef2332, Snef1252, Snef2550, Snef543, Snef1521, Snef1326, Snef1938, Snef2541, Snef102, Snef372, Snef1506, Snef2421, Snef2426, Snef244, Snef969, Snef2438, Snef2511, Snef21, Snef1536, Snef1421, Snef2373, Snef2397, Snef2533, Snef1317, Snef1116, Snef1460, Snef2348, Snef310, Snef384, Snef2271, Snef2507, Snef2264, Snef189, Snef606, Snef963, Snef763, Snef1154, Snef1518, Snef1757, Snef1558, Snef1249, Snef1510, Snef515, Snef816, Snef1156, Snef1292, Snef2330, Snef787, Snef2297, Snef2396 |
| 25.01-50.00 | Snef1489, Snef2462, Snef605, Snef651, Snef355, Snef81, Snef1320, Snef2486, Snef1278, Snef2555, Snef48, Snef945, Snef1162, Snef1644, Snef1758, Snef298, Snef899, Snef1305, Snef44, Snef683, Snef967, Snef2456, Snef417, Snef931, Snef951, Snef2339, Snef43, Snef2515, Snef412, Snef904, Snef1118, Snef1857, Snef2452, Snef73, Snef655, Snef1281, Snef134, Snef712, Snef1517, Snef27, Snef41, Snef921, Snef953, Snef1508, Snef2463, Snef387, Snef997, Snef2481, Snef149, Snef1332, Snef228, Snef1170, Snef1355, Snef2552, Snef1159, Snef2400, Snef2519, Snef1308 ,Snef12, Snef76, Snef302, Snef666, Snef978, Snef1023, Snef1030,, Snef1139, Snef1477, Snef2579, Snef2439, Snef798, Snef1157, Snef1242, Snef1679, Snef212, Snef374, Snef1505, Snef1748, Snef2307, Snef2425, Snef2077, Snef2333, Snef304, Snef29, Snef980, Snef89, Snef163, Snef976, Snef1294, Snef2443, Snef2508, Snef986, Snef1137, Snef1527, Snef1637, Snef2490, Snef317, Snef198, Snef836, Snef1567, Snef1868, Snef227, Snef1675, Snef988, Snef407, Snef1149, Snef1511, Snef155, Snef694, Snef1544, Snef508, Snef772, Snef1471, Snef206, Snef684, Snef87, Snef934, Snef1350, Snef1512, Snef1533, Snef2514, Snef852, Snef971, Snef2266, Snef2488, Snef488, Snef840, Snef1167, Snef2557, Snef1141, Snef505, Snef863, Snef1143, Snef1335, Snef1798, Snef1806, Snef2475, Snef2483, Snef960, Snef1762, Snef1794, Snef1889, Snef2527, Snef652, Snef1072, Snef1205, Snef1296, Snef1509, Snef2469, Snef2536, Snef218, Snef158, Snef509, Snef649, Snef992, Snef1440, Snef313, Snef1187, Snef947, Snef1196, Snef2329, Snef2340, Snef55, Snef248, Snef1513, Snef1571, Snef1863, Snef2293, Snef2494, Snef1420, Snef5, Snef324, Snef411, Snef518, Snef582, Snef638, Snef909, Snef1003, Snef1117, Snef1182, Snef1177, Snef1224, Snef1352, Snef1594, Snef1630, Snef2050, Snef2482, Snef2520, Snef1411, Snef128, Snef1323, Snef1756, Snef1866, Snef2464, Snef2513, Snef681, Snef1515, Snef2451, Snef2529, Snef2471, Snef511, Snef2267, Snef2278, Snef83, Snef1481, Snef1931, Snef1132, Snef1359, Snef1424, Snef613, Snef779, Snef898, Snef987, Snef1298, Snef1557, Snef1587, Snef1625, Snef1765, Snef1959, Snef278, Snef289, Snef82, Snef2074, Snef323, Snef338, Snef1129, Snef150, Snef1542, Snef1520, Snef2355, Snef2547, Snef142, Snef922, Snef1085, Snef311, Snef266, Snef608, Snef1251, Snef1253, Snef1306, Snef1606, Snef2526, Snef959, Snef1210, Snef23, Snef179, Snef299, Snef952, Snef1283, Snef1507, Snef1656, Snef2458, Snef2553, Snef849, Snef1638, Snef2459, Snef315, Snef365, Snef991, Snef1179, Snef1660, Snef1180, Snef1943, Snef2319, Snef677, Snef2604, Snef1267, Snef1219, Snef1316, Snef2467, Snef2630, Snef2537, Snef439, Snef1105, Snef1878, Snef503, Snef653, Snef1693, Snef2645, Snef383, Snef140, Snef679, Snef955, Snef1541, Snef1958, Snef1666, Snef156, Snef1498, Snef1777, Snef2473, Snef84, Snef1543, Snef2079, Snef364, Snef1464, Snef2395, Snef2055, Snef1302, Snef1531, Snef2551, Snef427, Snef841, Snef908, Snef965, Snef1060, Snef2282, Snef925, Snef880, Snef28, Snef301, Snef2272, Snef187, Snef243, Snef1530, Snef129, Snef1115, Snef1140, Snef144, Snef616, Snef942, Snef2450, Snef1417, Snef1548, Snef576, Snef673, Snef1496, Snef930, Snef177, Snef603, Snef1552, Snef1538, Snef1776, Snef2312, Snef2472, Snef2495, Snef353, Snef1621, Snef1270, Snef848, Snef1356, Snef180, Snef143, Snef1275, Snef1330, Snef1358, Snef1439, Snef1482, Snef2353, Snef1674, Snef2302, Snef2487, Snef910, Snef1313, Snef2457, Snef981 |
| 50.010-75.00 | Snef39, Snef199, Snef1128, Snef1711, Snef1764, Snef1022, Snef1046, Snef1897, Snef1153, Snef1631, Snef1805, Snef1166, Snef1785, Snef1932, Snef1525, Snef890, Snef1534, Snef385, Snef487, Snef1346, Snef1304, Snef2509, Snef609, Snef2523, Snef979, Snef1303, Snef1373, Snef2454, Snef2525, Snef2499, Snef2280, Snef358, Snef929, Snef1106, Snef1418, Snef118, Snef2356, Snef33, Snef489, Snef915, Snef1126, Snef1178, Snef277, Snef730, Snef996, Snef1065, Snef1185, Snef2504, Snef2588, Snef103, Snef1465, Snef104, Snef570, Snef785, Snef1163, Snef1945, Snef2659, Snef2665, Snef1221, Snef271, Snef647, Snef1096, Snef1549, Snef1783, Snef2532, Snef1054, Snef1362, Snef2522, Snef322, Snef486, Snef776, Snef1661, Snef108, Snef363, Snef2323, Snef519, Snef1100, Snef1612, Snef101, Snef687, Snef1490, Snef1657, Snef2430, Snef2455, Snef249, Snef1274, Snef1855, Snef615, Snef1215, Snef2788, Snef2516, Snef1949, Snef117, Snef449, Snef1476, Snef153, Snef661, Snef1127, Snef1595, Snef877, Snef1793, Snef2447, Snef2468, Snef1168, Snef314, Snef59, Snef204, Snef47, Snef53, Snef132, Snef178, Snef490, Snef1142, Snef1884, Snef2543, Snef110, Snef193, Snef127, Snef200, Snef69, Snef371, Snef1523, Snef1646, Snef650, Snef2465, Snef1120, Snef106, Snef438, Snef1775, Snef165, Snef856, Snef1494, Snef693, Snef897, Snef1133, Snef2259, Snef2270, Snef2383, Snef116, Snef151, Snef272, Snef604, Snef692, Snef2510, Snef633, Snef1232, Snef2309, Snef2498, Snef305, Snef1070, Snef2369, Snef186, Snef290, Snef523, Snef1935, Snef337, Snef1121, Snef1299, Snef1493, Snef1379, Snef2502, Snef135, Snef174, Snef303, Snef1560, Snef2428, Snef2505, Snef1183, Snef516, Snef618, Snef2501, Snef1551, Snef2315, Snef239, Snef329, Snef1632, Snef2372, Snef994, Snef2433, Snef36, Snef2255, Snef2289, Snef268, Snef263, Snef501, Snef663, Snef2378, Snef408, Snef2073, Snef932, Snef2385, Snef30, Snef49, Snef133, Snef139, Snef440, Snef676, Snef1203, Snef1225, Snef1314, Snef1911, Snef2294, Snef2370, Snef2445, Snef2517, Snef2493, Snef2528, Snef1501, Snef827, Snef1091, Snef2460, Snef466, Snef2301, Snef2654, Snef544, Snef627, Snef321, Snef513, Snef2234, Snef2476, Snef1204, Snef2362, Snef2342, Snef550, Snef1119, Snef1802, Snef2322, Snef42, Snef935, Snef1803, Snef2287, Snef2549, Snef1925, Snef190, Snef2350, Snef37, Snef507, Snef2388, Snef2474, Snef261, Snef279, Snef1055, Snef234, Snef24, Snef147, Snef370, Snef1948, Snef2521, Snef54, Snef195, Snef57, Snef194, Snef242, Snef2262, Snef2485, Snef20, Snef312, Snef962, Snef46, Snef205, Snef1184, Snef2313, Snef2477, Snef557, Snef2364, Snef203, Snef762, Snef917, Snef2327, Snef2538, Snef141, Snef209, Snef617, Snef1285, Snef361, Snef2283, Snef362, Snef728, Snef2354, Snef3, Snef148, Snef1295, Snef2349, Snef2351, Snef145, Snef2368, Snef799, Snef686, Snef968, Snef1504, Snef280, Snef889, Snef905, Snef115, Snef1186, Snef1893, Snef920, Snef126, Snef2053, Snef2274, Snef1188, Snef1338, Snef1918, Snef2331, Snef2057, Snef2357, Snef1360, Snef2338, Snef2253, Snef51, Snef85, Snef214, Snef504, Snef1581, Snef2453, Snef2466, Snef2530, Snef2256, Snef2328, Snef1778, Snef114, Snef414, Snef1449, Snef2285, Snef853, Snef359, Snef2269, Snef2310, Snef125, Snef157, Snef1049, Snef2311, Snef283, Snef2389, Snef2539, Snef2399, Snef1246, Snef40, Snef164, Snef173, Snef654, Snef659, Snef1255, Snef1488, Snef2257, Snef2361, Snef50, Snef52, Snef657, Snef1344, Snef1859, Snef2422, Snef2506, Snef502 |
| 75.01-100 | Snef270, Snef1124, Snef1101, Snef2298, Snef122, Snef884, Snef1528, Snef2386, Snef2393, Snef2503, Snef265, Snef2284, Snef38, Snef121, Snef907, Snef2335, Snef56, Snef2295, Snef2279, Snef183, Snef210, Snef247, Snef778, Snef1110, Snef26, Snef450, Snef1939, Snef434, Snef240, Snef240, Snef1322, Snef1891, Snef2292, Snef437, Snef944, Snef1138, Snef2043, Snef185, Snef333, Snef1348, Snef1865, Snef1873, Snef2300, Snef2437, Snef1852, Snef2345, Snef260, Snef1339, Snef1853, Snef2324, Snef581, Snef25, Snef1438, Snef2394, Snef363, Snef1854, Snef1851, Snef622, Snef2382, Snef2281, Snef354, Snef1883, Snef2367, Snef1910 |
